# Building Relations with Radiology Administrators

**DOI:** 10.3390/diagnostics7020033

**Published:** 2017-06-07

**Authors:** Megan Kalambo, Jay R. Parikh

**Affiliations:** Department of Radiology, MD Anderson Cancer Center, 1515 Holcombe Blvd—Unit 1350, Houston, TX 77030, USA; JRParikh@mdanderson.org

**Keywords:** administrator, manager, radiologist, conflict, dyad leadership

## Abstract

In some radiology departments, the lack of alignment between administrators and radiologists can pose significant challenges. This article describes how differences in background and priorities between administrators and radiologists can contribute to conflict and presents strategies on how to manage the conflict in a way that can leverage positive change. Strategies to build relations between radiologists and radiology administrators are described.

## 1. Introduction

Conflict exists in the workplace, irrespective of the job sector. Conflict often arises between co-workers, supervisors and subordinates, employees and customers when the involved parties perceive a threat to their needs, interests, or concerns [1,2,3,4,5,6]. In health care, jobs are heavily interdependent in the care of the patient. Healthy conflict can foster innovation and improved productivity while unhealthy conflict can stifle it. Poorly managed conflicts generate a breakdown in trust and lost productivity [1,7,8,9]. For a radiology department, the loss of trust and productivity have significant consequences that can directly impact the delivery of patient care [10,11].

In a radiology department, team members hold a range of skill sets, educational levels, salaries, and experience. The radiologist provides the professional expertise required to interpret the radiology exams but their work does not take place in a vacuum [12,13,14]. They are heavily dependent on the proficiency set of their technologists to obtain the appropriate images necessary for accurate and timely evaluation and diagnosis of their patients. In addition to the technologists, mid-level providers may also be involved in the care of patients. Physician assistants may be employed to help perform interventional procedures in the department. Nurses may also work with the radiologists in interventional procedures and examinations, especially those requiring conscious sedation. Schedulers and front desk staff play an integral role in the radiology department patient experience. In many imaging departments, clerical staff, technologists, and other support staff report to the radiology administrator who oversees the day to day operational functions of the practice.

In an environment where the radiologist group and radiology department staff do not share a common employer [15,16,17], an additional level of complexity is introduced into the departmental dynamics that may result in additional sources of conflict. In this setting, the radiologist may be employed by a private radiology group [14] or a partner institution [16,17]. The goal of this article is to educate our membership regarding how differential training and goals between administrators and radiologists may contribute to conflict, and to provide constructive strategies to leverage conflict in such a way to affect positive change.

## 2. Inherent Differences between Administrators and Physicians

A radiology administrator’s experience and training can vary from practice to practice. Most radiology administrators have a four-year degree and may hold a master’s degree or additional certification by their governing body such as the Radiology Administration Certification Commission. They oversee the day to day scheduling, workflow, finances, and equipment and often represent the department at various hospital meetings, community events, and professional meetings.

A detailed study with associated literature review from England [18] analyzed the differences between doctors and managers in goals, core values, codes of acceptable behavior, needs, and sources of frustration. Physicians were found to be highly driven by a desire to be respected and gain recognition by colleagues. The underpinning of their autonomy is that it is ‘earned’. Physicians have been trained in a culture of individualism and feel a high sense of responsibility for the patient. As a result, they typically resent system transparency, policies, and procedures. Physicians were shown to have strong needs for affiliation with their peers and for work life balance and tended to be more loyal to one facility or healthcare system.

In contrast, a manager’s desire for autonomy is often linked to feeling empowered to take risks and innovate within the organization. They are trained more in the environment of collectivism where their primary responsibilities are to the team, organization, and community [18]. Managers are relatively comfortable with transparency, policies, and procedures and tend to change organizations during a career to gain additional skill and receive promotions. Administrators have a need for achievement by attaining institutional targets.

It is critical for both radiologists and administrators to understand how differing values, needs, and desires can result in differing opinions surrounding a key issue. This diversity in opinion represents both an opportunity to address conflict and establish team growth [11,13].

## 3. Conflict Management

In this discussion of conflict, it is important to differentiate between the dynamics of a group versus team [2,7,8]. This becomes increasingly important in the setting of workplace morale and productivity. Groups are people who work together, while a team is a small group of people with similar skill sets that are committed to a common purpose [7,11]. Group members can often think that they are grouped together for administrative purposes only, while team members recognize their independence and understand both personal and team goals are best accomplished with mutual support. Group members tend to focus on themselves because they are not sufficiently involved in planning the unit’s objectives; team members feel a sense of ownership for their jobs and unit, because they are committed to value based common goals that they helped create [11]. Group members are often told what to do rather than being asked what they think the best approach would be. Team members are leveraged to actively contribute to the organizations success by applying their unique talents, knowledge, and creativity to team objectives. Simply, a team is a group that trusts each other. Teams that lack trust have difficulty in engaging in unfiltered passionate debate [3]. The ability to trust and engage in healthy conflict are fundamental for teams [19,20].

Several articles have been written regarding conflict management, particularly in the healthcare workplace [1,10,19,21,22]. The literature consistently underscores the inherent value of identifying the root cause of conflict and considers it a critical component of effective leadership [1]. Once the source of the conflict is understood, the most important next step is to properly address it. There are consequences for avoiding and/or mismanaging disagreements in the workplace and leadership should feel empowered to tackle and address these difficult conversations head on [20,23,24,25].

## 4. Alignment of Radiologist and Administrator

In attempting to bridge the differences between radiologists and administrators, it is important to identify areas where the two groups share common ground. Taylor et al. [18] demonstrated that physicians and managers are aligned in understanding that patient care is the primary concern and there is mutual regard for trust, honesty, and integrity. Both groups also share vested interest in improving systems and organizational infrastructure to ensure tasks are completed in an efficient and timely manner. The study also suggests that both groups share frustrations with bureaucracy, particularly in the setting of too many initiatives, not being listened to, and changes being imposed on them. Both groups like clear, attainable targets. Both groups need to be allowed to influence the changes that affect them.

These commonalities between the radiologists and the administrator represent an opportunity to establish common ground and mutual respect than can help lead teams in the radiology workplace. In transformational leadership for healthcare [26], leadership moves away from a traditional transactional top-down burning platform-type vision to a shared ambitious vision. Leadership is encouraged to develop organizations that are nimble, transparent, horizontal rather than hierarchical, and which empower their people to execute based on the concept of “shared consciousness”. Mechanisms that underlie transformational leadership are the four I’s—individualized consideration, intellectual stimulation, inspirational motivation, and idealized influence. Sharing the vision effectively can inspire staff, increasing overall morale and productivity [8,14,26].

## 5. The Dyad Leadership Model as a Tool

The dyad model of leadership represents a potential relationship bridge between radiologist leadership and their administrative counterparts, and may help effectively address conflict between an administrator and radiologists which may arise from lack of effective communication [27]. Historically, most business settings have worked in the context of a traditional operational model. Typically, directors, managers, and supervisors work under the hierarchical leadership of an executive administrator, such as a vice-president. In this pyramidal structure, typically supervisors report to managers, who in turn report to directors, who in turn report to vice presidents. The strengths of this leadership model are the unambiguous authority and direct decision-making. Organizational initiatives are typically efficiently implemented, as subordinates are not empowered to reject direction. A critical drawback of this model in healthcare settings is that radiologists, except for historically established positions such as chief medical officers, typically do not take on leadership roles. With lack of physician engagement, it is difficult to build trust [3,28].

The definition of the dyad model varies across health systems, but at its core, the operations arm is partnered at every level in the organization with a physician leader, including radiology. With inherent radiologist engagement, mistrust is reduced [28,29,30]. It is often easier to implement new imaging protocols and/or standardized workflows with a radiologist champion [31]. This infrastructure heavily relies on the identification of radiologists with respected clinical experience that demonstrate leadership skills and possess an understanding of the business [8,21,32]. For the dyad to succeed, the business knowledge and leadership of the administrator will need to be reciprocally harvested and respected by the physician partner.

## 6. Termination

In cases where there is continued conflict despite attempts at intervention, termination of the administrator may be pursued. It is important to assert that termination by the employer should only be executed after all interventions and efforts have been completely exhausted. Often, there are additional concerns from staff and other administrators that contribute to the decision for termination. This outcome should be understood as a significant loss by all involved parties.

There are both direct and indirect costs with termination. Direct expenses of conflict include litigation costs, management productivity losses secondary to involvement in conflict resolution rather than performing administrative tasks, turnover costs for training new staff, disability/stress claims, as well as possible sabotage, theft, and damage to facilities by those involved with the conflict [1,11]. Perhaps more significant expenses are related to the indirect costs related to reduced team morale, decreased patient satisfaction, compromised patient safety issues, tarnished reputation of the health care organization, disrespect for the radiologist(s), and emotional costs directly involved in the conflict [1,11]. Following the announcement, the radiologists at the center should conduct themselves professionally. There is no cause for celebration in the context of emotional trauma; such behavior will reduce the respect and status of the radiologists by the remainder of the hospital leadership team and staff. Radiologists should acknowledge the loss, inform senior administration that they are committed to creating a positive environment in the workplace, and would like to participate as players in the recruitment of the next administrator. In summary, radiologists should demonstrate character and resolve in the face of adversity.

## 7. Conclusions

In some radiology departments, the lack of alignment between administrators and radiologists can post significant challenges. The inevitability of conflict in the workplace requires that those in a position of leadership possess the appropriate training to help navigate, manage, and resolve differences [11,29]. Given the multifaceted dynamics inherent in a radiology practice, shared governance between the radiologists and the practice administrators can help create a work environment with a unified vision, sense of employee ownership, and mutual understanding. This dyad model of leadership can also assist in establishing radiology practice standards for communication, engagement, and performance that optimize the success of the radiology team. Building an effective radiology team can lead to improved morale, job satisfaction, enhanced patient care, and an overall increase in department productivity.

## References

[1] Harolds J., Wood B.P. (2006). Conflict management and resolution. J. Am. Coll. Radiol..

[2] Simpao A.F. (2013). Conflict management in the health care workplace. Physician Exec..

[3] Tiffan B. (2009). Dealing with difficult people. Physician Exec..

[4] Rosenstein A.H. (2011). Disruptive physician behavior. Strategies for addressing the cause and effect. Healthc. Exec..

[5] Rosenstein A.H. (2015). Physician disruptive behaviors: Five year progress report. World J. Clin. Cases.

[6] Hertz B.T. (2015). Managing conflict with patients. Effective communication is vital to managing disagreements with patients to resolve tension and prevent negative outcomes. Med. Econ..

[7] Toegel G., Barsoux J.L. (2016). How to Preempt Team Conflict. Harv. Bus. Rev..

[8] Haas M., Mortensen M. (2016). The Secrets of Great Teamwork. Harv. Bus. Rev..

[9] Ulreich S., Harris R.D., Sze G., Moriarity A.K., Bluth E. (2015). The disruptive radiologist. J. Am. Coll. Radiol..

[10] Sportsman S., Hamilton P. (2007). Conflict management styles in the health professions. J. Prof. Nurs..

[11] Huckman R., Staats B. (2013). The hidden benefits of keeping teams intact. Harv. Bus. Rev..

[12] Gunderman R.B., Weinreb J.C., van Moore A., Hillman B.J., Neiman H.L., Thrall J.H. (2008). Radiology practice models: The 2008 ACR Forum. J. Am. Coll. Radiol..

[13] Gunderman R.B., Saravanan A. (2010). From conflict to collaboration. J. Am. Coll. Radiol..

[14] Muroff L.R. (2004). Implementing an effective organization and governance structure for a radiology practice. J. Am. Coll. Radiol..

[15] Afable R., Brant-Zawadzki M.N. (2013). Changing radiology in the changing hospital environment. J. Am. Coll. Radiol..

[16] Natesan R., Yang W.T., Tannir H., Parikh J. (2016). Strategic Expansion Models in Academic Radiology. J. Am. Coll. Radiol..

[17] Parikh J.R., Brown J., Yang W.T., Tannir H. (2017). Network Collaboration of an Academic Institution and a Community Health Organization. J. Am. Coll. Radiol..

[18] Taylor H., Benton S. (2008). The doctor-manager relationship: A behavioural barrier to effective health care?. Stud. Health Technol. Inform..

[19] Harolds J. (2005). Effective radiology teams. J. Am. Coll. Radiol..

[20] Rosenstein A.H., Dinklin S.P., Munro J. (2014). Conflict resolution: Unlocking the key to success. Nurs. Manag..

[21] Harolds J. (2004). Selected important characteristics for enlightened medical leaders. J. Am. Coll. Radiol..

[22] Kayser J.B. (2015). Mediation Training for the Physician: Expanding the Communication Toolkit to Manage Conflict. J. Clin. Ethics.

[23] Muroff L.R. (2008). Taking your radiology practice to the next level. J. Am. Coll. Radiol..

[24] Rosenstein A.H. (2012). Physician communication and care management: The good, the bad and the ugly. Physician Exec..

[25] O’Connor E.J., Liebscher L.A., Fiol C.M. (2004). Beating them or joining them: Your radiology group’s path to the future. J. Am. Coll. Radiol..

[26] Bass B.M. (1990). From transactional to transformational leadership: Learning to share the vision. Organ. Dyn..

[27] Baldwin K.S., Dimunation N., Alexander J. (2011). Health care leadership and the dyad model. Physician Exec..

[28] Rosenstein A.H. (2015). Strategies to Enhance Physician Engagement. J. Med. Pract. Manag..

[29] Tiffan B. (2011). The art of team leadership. Physician Exec..

[30] Shanafelt T.D., Gorringe G., Menaker R., Storz K.A., Reeves D., Buskirk S.J., Sloan J.A., Swensen S.J. (2015). Impact of organizational leadership on physician burnout and satisfaction. Mayo Clin. Proc..

[31] Kalambo M., Parikh J.R. (2017). Implementing Standardized Protocols During Geographic Radiology Expansion. J. Am. Coll. Radiol..

[32] Parikh J., Einstein A. (2006). Medical directors of breast imaging centers: Beyond films. J. Am. Coll. Radiol..

